# Subconjunctival 0.1% epinephrine versus placebo in maintenance of mydriasis during vitrectomy: a randomized controlled trial

**DOI:** 10.1186/s40942-018-0142-y

**Published:** 2018-10-17

**Authors:** Rafael B. de Araújo, Breno M. S. Azevedo, Thais S. Andrade, Maria F. Abalem, Mário L. R. Monteiro, Pedro C. Carricondo

**Affiliations:** 1 Division of Ophthalmology, Hospital Universitário Onofre Lopes, Universidade Federal do Rio Grande do Norte, Rua Mipibu, 741 apt 1402A, Natal, RN 59014-480 Brazil; 2 Division of Ophthalmology, Hospital das Clínicas HCFMUSP, Universidade de Sao Paulo, Rua Dr. Ovídio Pires de Campos, 225, 05403-010 São Paulo, SP Brazil

**Keywords:** Epinephrine, Pupil, Mydriasis, Vitrectomy

## Abstract

**Background:**

Pupil dilation and mydriasis maintenance throughout vitreoretinal surgeries are important to allow satisfactory fundus visualization and reduce risk of complications. The purpose of this study is to evaluate the role of subconjunctival epinephrine 0.1% injection in mydriasis maintenance during vitrectomy.

**Methods:**

Ninety-nine consecutive patients undergoing vitrectomy were enrolled. All subjects were preoperatively dilated with tropicamide 1%. Each patient was randomly allocated either in the epinephrine or placebo group. In epinephrine group, patients were submitted to a 0.2 cc subconjunctival injection of a 0.1% epinephrine solution just before first incisions. In placebo group, the same procedure was performed with 0.2 cc of saline 0.9%. Horizontal pupil diameter was measured with calipers before and in the end of the procedure.

**Results:**

Patients in the epinephrine group showed a significantly larger mean pupil diameter in the end of the surgery compared to placebo. There was a significant increase of mean pupil diameter from the beginning to the end of the surgery in such patients. Blood pressure was significantly higher in the epinephrine group than in placebo group. No other adverse effects were noted.

**Conclusion:**

Subconjunctival epinephrine is effective for maintaining and increasing pupil size during vitrectomy, compared to placebo. Caution should be taken regarding intraoperative blood pressure levels.

**Trial registration:**

RBR; RBR-3qzhvg; Registered 8 May 2018—Retrospectively registered, http://www.ensaiosclinicos.gov.br/rg/RBR-3qzhvg/.

## Background

Proper mydriasis is paramount for satisfactory view of the retinal fundus during pars plana vitrectomy (PPV). Pupil dilation is usually obtained by preoperative instillation of mydriatic drops, providing appropriate view of the posterior segment in most cases. However, there has been demonstrated that in prolonged PPVs a prostaglandin mediated miosis secondary to the surgical trauma becomes clinically important, raising the risk of complications such as incomplete vitreous removal, lens touching and traumatic membrane peeling [1].

Epinephrine is a widely used mydriatic agent, acting both by contracting the dilator iris muscle by its alfa receptor actions and relaxing the sphincter by its beta effects [2]. The use of intracameral epinephrine for intraoperative pupil dilation is a common practice among cataract surgeons, with satisfactory efficacy and safety [3]. In case of PPV, it is preferable to avoid anterior chamber manipulation unless strictly necessary. In addition to an extra corneal incision, the intracameral approach could result in further iris trauma and pupil miosis and instability of anterior chamber, especially when using contact lenses visualization systems. [4, 5].

Several mydriatic combinations have been tested for both intracameral and subconjunctival use to maintain pupil size during phacoemulsification [6–9], but only a few strategies have been demonstrated to achieve and maintain mydriasis during PPV and no previous study have tested the efficacy of subconjunctival epinephrine alone for this purpose. This study compared the efficacy and side effects of pupil dilation and its maintenance during PPV between patients who had subconjunctival injection of 0.2 cc of epinephrine (1:1000) and patients who had saline 0.9% prior to surgery.

## Methods

This randomized, placebo-controlled trial was performed at the Hospital das Clínicas, University of Sao Paulo. The study was approved by the local ethics review board and conducted according to the Declaration of Helsinki. Informed consent was obtained from all patients prior enrollment.

We included 99 consecutive patients who underwent PPV. Indications for PPV included rhegmatogenous and tractional retinal detachment, vitreous hemorrhage, macular hole and epiretinal membrane. Pseudophakic patients were included if the cataract surgery had been performed without complication at least 6 months prior to randomization. Exclusion criterion included past history of uveitis, pseudoexfoliation, trauma, use of any systemic or topical anti-inflammatory medications within 2 weeks prior to surgery, or intraoperative complications. Patients submitted to scleral buckling or any anterior chamber procedure during PPV were also excluded.

All patients were dilated with 3 consecutive drops of tropicamide 1%, 10 min apart, 30 min prior to surgery. Pupil diameter was measured at the three time points: baseline, prior to dilation on slit lamp examination; at the beginning of the surgery, prior to the first incision under microscope visualization with an eyelid speculum and a 0.5 mm accuracy caliper (preoperative); and at the end of the surgery. The main outcome measure was the horizontal diameter noted in millimeters (mm). Following the pupil measurement, patients received either a 0.2 cc subconjunctival injection of epinephrine 1:1000 (study group) or saline solution 0.9% (control group) in the inferonasal perilimbic area. Age, sex, iris color (dark versus light), diagnosis of systemic hypertension, mean arterial blood pressure (MABP) at the beginning and at the end of the procedure, preoperative blood sugar level and duration of the surgery were obtained and compared between groups.

Data were summarized numerically with counts and percentages, means and standard deviations (SD). Kolmogorov–Smirnov test was performed to test for the normality assumption. The two-sample Student’s *t* test and Pearson Chi square test were performed to compare continuous and nominal variables, respectively. All tests were performed at 0.05. Statistical analysis was performed by SPSS (ver. 23.0; SPSS, Chicago, USA).

## Results

Forty-five patients were allocated in the study group and 44 were allocated in the control group. There was no statistically significant difference between the two groups for duration of surgery, age, gender, iris color, presence of systemic hypertension, blood sugar level and MABP, both at the beginning and at the end of the procedure. Table 1 shows the descriptive and demographic profiles of patients from the study and controls groups.

**Table 1:** Demographic and descriptive data of patients from study and control groups

Variable	Study	Control	*P*
N	45	44	
Age (years)	63.7 (± 14.6)	57.9 (± 14.1)	0.253^a^
Sex (male/female)	27/18	21/23	0.291^b^
Light/dark irides	9/36	9/35	0.583^b^
Systemic hipertension	48.9%	59.0%	0.397^b^
Procedure time (min)	50.3 (± 20.7)	49.5 (± 23.0)	0.911^a^
Blood sugar level (mg/dL)	129.7 (± 42.5)	116.8 (± 36.4)	0.359^a^
Preoperative MABP (mmHg)	98.9 (± 11.9)	102.9 (± 12.8)	0.114^a^
Posoperative MABP (mmHg)	104.8 (± 10.2)	105.7 (± 8.2)	0.629^a^

*MAPB* mean arterial blood pressure, *Min* minutes

^a^Two-sample Student’s *t* test. Values expressed in means (± standard deviation)

^b^Pearson Chi square test

There was an increase of MABP at the end of the surgery compared to the beginning of the surgery, with a larger variation in the study group. Paired *t* test showed a significant statistically difference between final and initial MAPB in the study group (*p* = 0.001), whereas no significant statistically differences were observed in the control group (*p* = 0.086). No adverse events were observed in patients from neither group.

The mean pupil diameters of both groups at the three time points are demonstrated in Table 2. The mean pupil diameter was 2.9 mm in both groups at baseline (p = 0.902). There were no statistically significant differences between both groups neither in the baseline nor in the beginning of the surgery. However, the mean pupil diameter at the end of the surgery was larger (7.3 mm) in the study group compared to the control group (6.2 mm) (p < 0.001).

**Table 2:** Mean pupil diameter at baseline, at the beginning and at the end of surgery of both study and control groups

Measurements	Study	Control	P*
Baseline	2.9 (± 0.6)	2.9 (± 0.4)	0.902
Beginning of surgery	6.7 (± 1.1)	6.5 (± 1.0)	0.619
End of surgery	7.3 (± 0.6)	6.2 (± 0.9)	< *0.001*

*Student’s *t* test. Significant values in italic

In addition, there was an increase of the mean pupil diameter at the end of the surgery compared to the beginning of the surgery in patients from the study group. In contrast, the control group had a smaller mean pupil diameter at the end of the surgery, compared to the beginning of the surgery.

## Discussion

This study evaluated the use of subconjunctival epinephrine for mydriasis maintenance during PPV. Our findings indicated that a subconjunctival administration of epinephrine at the beginning of the surgery not only led to an appropriate pupil size during PPV as it increased the mean pupil size from the beginning (6.7 ± 1.1 mm) to the end of the surgery (7.3 ± 0.7 mm). Our study agrees with a previous study by Kulshrestha et al. [10] that found that subconjunctival injections of mydricaine, a mixture of atropine, epinephrine and procaine, were useful in mydriasis maintenance during vitrectomy when associated to preoperative topical diclofenac. This study, however, did not have a placebo-controlled group.

Intraoperative miosis is believed to be mediated by prostaglandins released from the uveal vasculature [11]. This rationale has been used by several authors for testing non-steroidal anti-inflammatory agents for mydriasis maintenance [12–16], especially in cataract surgery. Although our study did not compare subconjuntival epinephrine with any anti-inflammatory agent, Jha et al. [17] demonstrated that the use of intracameral nepafenac had no impact on intraoperative pupil diameter of rabbit eyes. Other previous studies also showed no advantage in preventing intraoperative miosis in patients undergoing vitreoretinal surgery by using topical flurbiprofen [1, 18].

A myriad of strategies have been used for mydriasis achievement and maintenance during phacoemulsification with topical [19], intracameral [9, 13] or subconjunctival [10] administration of mydriatic drugs, soaked sponges [20] or slow release drug inserts [21]. Intracameral epinephrine has been the most widely strategy used by cataract surgeons, with several reports of its efficacy and safety [3, 22–24]. Theoretically, epinephrine infused into the eye can potentially be systemically absorbed both via vascular structures of the anterior segment, like scleral and conjunctival vessels, and via nasolacrimal duct [3]. In the present study, there were no significant changes in heart rate between control and study groups, but there was a statistically significant increase on MABP from the beginning to the end of the procedure in patients receiving subconjunctival epinephrine. There has been described in previous case reports with mydricaine use for mydriasis maintenance during vitrectomy the possibility of adverse cardiac events, such as isolated sinus tachycardia [25, 26], myocardial ischemia [27, 28] and atrial fibrillation [29]. Nevertheless, the increase in MAPB levels did not need to be controlled by any further medication and were considered clinically non-significant by the anesthesiologists in all patients. No other systemic adverse events were observed in our patients.

In our study the age, sex, irides color, procedure duration, history of systemic hypertension, blood pressure and preoperative blood sugar levels were similar in both groups. Therefore, this study was unable to evaluate the impact of these variables on such intervention. Although the mean procedure duration in our study was long (approximately 50 min) in both groups, our results may not be extrapolable for longer surgeries. In addition, this study excluded patients undergoing associated scleral buckling procedures, to avoid bias related to the subconjunctival absorption, once perilimbic peritomy is performed in such cases. Thus, it is unknown whether the use of subconjunctival epinephrine is suitable for those patients.

## Conclusions

In conclusion, subconjunctival administration of epinephrine appears to be an effective strategy to achieve and maintain mydryasis during PPV without the need to manipulate the anterior chamber and possibly avoiding related risks, such as endothelial injury, lens touching and corneal incision leakage. This strategy seems to be safe, but caution still should be taken about blood pressure levels. Further studies are necessary to assess the combination of subconjunctival epinephrine with topical ocular anti-inflammatory agents and to better investigation of possible long-term systemic and ocular adverse effects.

## Abbreviations

MABP: mean arterial blood pressure
SD: standard deviation
